# Wasted Food: U.S. Consumers' Reported Awareness, Attitudes, and Behaviors

**DOI:** 10.1371/journal.pone.0127881

**Published:** 2015-06-10

**Authors:** Roni A. Neff, Marie L. Spiker, Patricia L. Truant

**Affiliations:** 1 Department of Environmental Health Sciences, Johns Hopkins Bloomberg School of Public Health, Baltimore, Maryland, United States of America; 2 Center for a Livable Future, The Johns Hopkins University, Baltimore, Maryland, United States of America; 3 Program in Human Nutrition, Department of International Health, Johns Hopkins Bloomberg School of Public Health, Baltimore, Maryland, United States of America; 4 Department of Health Policy and Management. Johns Hopkins Bloomberg School of Public Health, Baltimore, Maryland, United States of America; Indiana University, UNITED STATES

## Abstract

The U.S. wastes 31 to 40% of its post-harvest food supply, with a substantial portion of this waste occurring at the consumer level. Globally, interventions to address wasted food have proliferated, but efforts are in their infancy in the U.S. To inform these efforts and provide baseline data to track change, we performed a survey of U.S. consumer awareness, attitudes and behaviors related to wasted food. The survey was administered online to members of a nationally representative panel (N=1010), and post-survey weights were applied. The survey found widespread (self-reported) awareness of wasted food as an issue, efforts to reduce it, and knowledge about how to do so, plus moderately frequent performance of waste-reducing behaviors. Three-quarters of respondents said they discard less food than the average American. The leading motivations for waste reduction were saving money and setting an example for children, with environmental concerns ranked last. The most common reasons given for discarding food were concern about foodborne illness and a desire to eat only the freshest food. In some cases there were modest differences based on age, parental status, and income, but no differences were found by race, education, rural/urban residence or other demographic factors. Respondents recommended ways retailers and restaurants could help reduce waste. This is the first nationally representative consumer survey focused on wasted food in the U.S. It provides insight into U.S. consumers’ perceptions related to wasted food, and comparisons to existing literature. The findings suggest approaches including recognizing that many consumers perceive themselves as being already-knowledgeable and engaged, framing messages to focus on budgets, and modifying existing messages about food freshness and aesthetics. This research also suggests opportunities to shift retail and restaurant practice, and identifies critical research gaps.

## Introduction

In the U.S., 31 to 40% of the U.S. post-harvest food supply goes to waste[1,2]. A substantial portion of this waste occurs at the consumer level, driven not only by consumer behaviors but also by practices at the processing, retail and restaurant levels and by broader social and economic factors[3,4]. This waste has immense consequences.

The lost nutritional value of post-harvest waste in the U.S. represents an estimated 1,249 calories per capita per day, with the greatest amount by weight coming from fruits and vegetables [1]. Waste impacts public, food industry and household budgets; food lost from harvest to consumer in 2010 cost $161.6 billion; losses at the consumer level averaged $371 per capita, or 9.2% of average food spending [1]. Addressing wasted food puts that food and/or money back into circulation, potentially contributing to improved nutrition and, among those with lower incomes, improved food security. More broadly, reducing waste could help offset the 60% increase in food the United Nations projects we will need from 2009 to 2050[5]. Because wasting food means wasting all the food’s “embodied” social and environmental impacts, this loss contributes extensive water, air and soil contamination [6] and harm to workers[7]. Wasted food in North America/Oceania also accounts for an estimated 35% of freshwater consumption, 31% of cropland, and 30% of fertilizer usage[8]; as well as 2% of U.S. greenhouse gas emissions[9]; and 21% of post-recycling municipal solid waste[10]. The avoidable use of limited resources and additional environmental impacts from wasted food contribute to the challenge of providing a sustainable and affordable food supply for the future.

While well-supported efforts to understand and address wasted food have proliferated around the world[11–13], in the U.S. these efforts are nascent, piecemeal, and primarily entrepreneurial, though there are some federal and state interventions[14,15]. Intensive, multifaceted efforts supported by research can work; for example, following a range of activities, the U.K. achieved a 21% reduction in avoidable consumer food waste in five years[16].

As prevention efforts ramp up in the U.S., there is a need for evidence to inform the approaches taken, as well as baseline data to assist in tracking progress. We performed a nationally representative consumer survey aimed at addressing research questions including:

- How aware are Americans of wasted food generally, and of food they waste?
- What attitudes shape their decisions about purchasing and discarding food?
- What would motivate them to waste less?
- To what extent do they perform behaviors known to increase or decrease waste?
- What retail and restaurant industry actions to reduce consumer-level waste of food are supported by consumers?

Surveys in several countries have addressed these topics, but due to differences in culture and society, food system, infrastructure, policy, and geography, we cannot presume how these findings might translate to the U.S. context. This survey identifies points of similarity and difference with that work, and highlights areas in need of additional in-depth research.

We define “wasted food” per the US Department of Agriculture (USDA) as “reductions in *edible* food mass anywhere along the food chain.” [17] (We prefer the term, “wasted food” to “food waste,” emphasizing that the item is essentially food rather than essentially waste.) For the purposes of the survey, in most cases, we used terms such as “discarding food” rather than “wasting food,” to reduce implied judgment and thus reduce bias in responses.

## Materials and Methods

### Survey Development

We designed a survey instrument to examine consumer awareness, knowledge, attitudes, and behaviors related to wasted food. To enable comparison, many of the questions were replicated from other surveys; in some cases the number of response options was reduced, or questions were edited for U.S. language usage or clarity [18–20]. Additional questions were added to expand upon topics of interest. The survey does not aim to quantify the amount of food consumers actually waste (surveys are inappropriate tools for measuring waste) but does ask qualitative questions about waste quantity. One section requests information about respondent performance of a set of behaviors we characterize as waste-promoting and waste reducing. We based these classifications on evidence summarized in literature from the U.K. and Australia [3,21,22]. Most of the demographic information was gathered in advance from panel participants by the GFK Knowledgeworks firm. The survey was administered online, enabling randomization of response option order for questions with multiple options. The survey instrument was reviewed by more than 20 colleagues, including those engaged in research and communications on wasted food, and survey design experts from our network and the firm, GFK Knowledgeworks, which administered the survey. The firm prepared a user-friendly interface reflecting principles of online survey design. The questionnaire is provided in S1 File.

### Survey Sampling and Implementation

GFK/Knowledgeworks maintains a nationally representative online panel with members randomly recruited using probability-based geographic criteria[23]. A key benefit of this approach is that it “covers” 97% of U.S. households in sampling nomenclature, regardless of whether they have cell phones, landlines or neither. To improve representation in its panel, GFK oversamples census blocks with high concentrations of African American and Hispanic residents, and provides Internet access and devices to those lacking them. The firm collects extensive demographic and background data on participants, supporting its ability to create survey samples that are representative of the U.S. non-institutionalized population. For sample selection purposes, the firm applies an adjustment based on the updated national demographic distribution for nine demographic variables. Following survey administration, the firm also supplies a set of post-stratification weights for use in analysis, based on benchmark distributions of seven demographic variables. GFK runs a modest incentive program including raffles or sweepstakes for cash and prizes; this program encourages participation in general, and is not linked to specific surveys.

The survey was formally piloted for two days with a random sample of respondents, and further modifications were made. The survey was administered from April 16 to 20, 2014, to a nationally representative sample of 1,998 non-institutionalized adults ages 18 and above. Reminders were sent on day 3. The response rate was 51%, yielding a sample size of 1,010 respondents.

### Analysis

Results were analyzed in Stata (version 13.1). We used chi-square tests of independence to test for associations with demographic variables, with statistical significance determined by p<0.05. Demographic variables reported here include gender, age (“older respondents” referring to those age 65 and above, and “younger respondents” referring to those under age 65”), parental status (“parents” referring to respondents with a child age 18 or under in the household), education (less than high school, high school, some college, or completed college), and household income quintiles (less than $29,999; $30,000 to $59,999; $60,000 to $84,999; $85,000 to $124,999; and $125,000 or more). Other demographic variables, such as household size and employment status, were not significantly associated with outcomes of interest reported here. In chi-square analyses, some categorical dependent variables were analyzed as binary variables so that results would be more readily interpretable. A future multivariate analysis will describe non-demographic predictors of awareness, attitudes and behaviors related to wasted food. Survey data may be found in S2 File.

### Ethics Statement

This study was reviewed by the Johns Hopkins Bloomberg School of Public Health Institutional Review Board (IRB), which determined it non-human subjects research. All subjects had previously consented to participate in GFK Knowledgeworks surveys. Due to the non-sensitive nature of the research and subject anonymity, the IRB determined there was not a need for additional consent procedures.

## Results

Table 1 describes the characteristics of the unweighted sample of 1,010 respondents. Survey weights were applied to further improve the sample’s representativeness, and are used in all analyses. Table 2 displays results of the chi-square analyses.

**Table 1:** Respondent Demographics, Unweighted^+^.

	Survey %	U.S. %
**Gender** (1)		
Male	50	49
Female	50	51
**Age** (1)		
18–24	7	14^a^
25–44	28	35 ^a^
45–64	40	35 ^a^
65 and older	25	18 ^a^
**Education** (2)		
Less than high school	11	12 ^a^
High school	32	30 ^a^
Some college	25	19 ^a^
College graduate	33	39 ^a^
**Race** (3)		
White, non-Hispanic	75	63
Black, non-Hispanic	8	13
Other, non-Hispanic	4	9
Hispanic	10	17
**Household Income** (3)		
<$19,999	12	19
$20,000—$39,999	20	20
$40,000—$59,999	17	17
$60,000—$99,0999	23	22
> $100,000	29	22
**Live with child/ren < 18** (2)	21	32 ^b^
**Self or parent is immigrant** (1)	17	13% ^c^ (self; NA for parents)

^**+**^Due to rounding, some categories do not sum to 100 percent.

^a^ percentage is based on population age 18, not total population.

^b^ refers to percentage of households with members under age 18

^c^ refers to percentage of foreign-born individuals

Sources for US data: 1–2012 CPS ASEC; 2–2014 CPS ASEC; 3–2013 CPS ASEC.

[24–26]

**Table 2:** Survey results (selected) and chi-square tests ^a^.

	ALL	Age		Gender		Child/ren under 18 in Household		Household Income Quintile ^b^					Highest Educational Attainment			
		Under 65	65 or Older	Female	Male	Yes	No	Q1	Q2	Q3	Q4	Q5	<High School	High School	Some College	College Graduate
**Perceived** **knowledge** **about reducing waste of food**																
Very knowledgeable	24%	23%*	30%*	24%	24%	13%*	27%*	26%	27%	22%	20%	23%	25%	22%	27%	22%
Not very, somewhat, or fairly	76%	77%*	70%*	76%	76%	87%*	73%*	74%	73%	78%	80%	77%	75%	78%	73%	78%
**Estimated** **U.S. waste of food**																
5 or 10%	10%	10%	11%	10%*	10%*	13%	9%	15%*	7%*	7%*	9%*	12%*	17%*	7%*	8%*	12%*
20%	33%	33%	35%	32%*	35%*	29%	35%	26%*	27%*	45%*	36%*	41%*	24%*	32%*	35%*	36%*
40%	45%	45%	43%	42%*	47%*	43%	45%	37%*	52%*	43%*	47%*	41%*	39%*	47%*	43%*	46%*
60%	12%	12%	11%	16%*	8%*	15%	11%	23%*	14%*	5%*	5%*	5%*	19%*	14%*	14%*	6%*
**Estimated** **household food discards**																
0%	13%	12%*	20%*	12%	14%	6%*	15%*	15%	14%	17%	9%	13%	18%	11%	13%	13%
10%	56%	55%*	59%*	53%	59%	56%*	56%*	51%	58%	50%	62%	56%	51%	56%	54%	59%
20%	21%	22%*	17%*	24%	19%	26%*	20%*	24%	16%	21%	22%	21%	16%	22%	25%	19%
30%	10%	11%*	5%*	11%	8%	12%*	9%*	10%	12%	11%	2%	10%	15%	11%	8%	9%
**Estimated household waste of food compared to the** **average American**																
More	3%	3%*	2%*	4%*	2%*	7%*	2%*	4%*	2%*	3%*	2%*	6%*	4%	2%	3%	4%
The same	24%	26%*	15%*	27%*	20%*	27%*	23%*	28%*	21%*	16%*	20%*	35%*	30%	24%	25%	20%
Less	73%	71%*	84%*	69%*	78%*	66%*	75%*	68%*	77%*	80%*	77%*	59%*	66%	74%	73%	76%
**Acceptance of** **brown banana**																
0–24%	35%	35%	36%	40%*	30%*	31%	37%	47%*	33%*	34%*	27%*	34%*	46%	38%	29%	34%
25–49%	32%	32%	33%	30%*	35%*	37%	31%	26%*	36%*	30%*	40%*	28%*	29%	31%	35%	33%
50–74%	19%	19%	17%	18%*	19%*	22%	18%	13%*	17%*	25%*	22%*	19%*	12%	19%	20%	20%
75–100%	14%	14%	14%	12%*	16%*	10%	15%	14%*	14%*	11%*	11%*	19%*	13%	12%	16%	13%
**Perceived** **current effort** **to minimize waste of food**																
A lot of current effort	35%	33%*	47%*	36%	34%	38%*	28%*	43%	37%	30%	32%	35%	37%	35%	39%	32%
None, a little, or a medium amount	65%	67%*	53%*	64%	66%	62%*	72%*	57%	63%	70%	68%	69%	63%	65%	61%	68%

* p<0.05

^a^ For each chi-square test, the percentages shown represent column proportions.

^b^ Household Income Quintiles: Q1: less than $29,000; Q2: $30,000 to $59,999; Q3: $60,000 to $84,999; Q4: $85,000 to $124,999; Q5: $125,000 or more.

### Awareness and Knowledge

The survey assessed respondents’ reported levels of awareness and knowledge about wasted food. In the past year, 42% indicated they had seen or heard information about wasted food and 16% had sought information about reducing it. In describing their knowledge about how to reduce the amount of food they discard, 24% described themselves as “very knowledgeable” and 38% described themselves as “fairly knowledgeable.” Age and parental status were significantly associated with self-reported knowledge: 30% of older respondents reported being “very knowledgeable,” compared to only 23% of younger respondents. Among non-parents, 27% felt “very knowledgeable,” compared to only 13% of parents (Table 2). S1 Fig describes additional information respondents would like to help reduce food discards.

As another gauge of awareness, respondents were asked to estimate the percentage of food in the US that is discarded or otherwise not eaten by humans. Media stories about wasted food commonly share Hall et al.’s (2009) estimate that 40% of the nation’s post-harvest food supply is wasted. As shown in Table 2, 45% of respondents provided this 40% figure, with most others providing lower estimates. Estimates of average household food waste in the U.S. were significantly associated with gender, household income quintile, and education.

Respondents were also asked to estimate the total percentage of food they themselves discard. While these estimates should not be taken literally, they are useful for gaining insight into how Americans perceive their waste levels and for comparing with evidence-based averages and perceptions about national waste. Table 2 shows that respondents overwhelmingly reported discarding low percentages of food they purchase, with 13% reporting that they did not discard any food, and 56% indicating they discarded 10% of purchased food. Only 10% said they discarded 30% or more. Age and parental status were significantly, but non-linearly associated with self-reported estimates of household discards (Table 2).

When asked to compare the amount of food they discard to that of others, 73% of respondents reported that they discard less than the average American household, and only 3% reported that they discard more. These comparisons were significantly associated with demographic factors including age, gender, parental status, and household income quintile, as shown in Table 2.

Respondents were asked to estimate qualitatively how much of their household’s food discarding could be avoided; 29% reported that “a fair amount” or “a lot” was avoidable. The only significant demographic association was with age; 10% of younger respondents reported that “a lot” of discarding was avoidable, versus only 3% of older respondents.

Qualitative ratings were also requested regarding the amount of food discarded in each of six possible categories. Respondents perceived themselves as throwing out the highest amount of fruits and vegetables, followed by homemade meals, bread, meat, milk, and packaged foods. Strikingly, 37% saw themselves as throwing out either “none” or “hardly any” food in all six categories.

### Attitudes and Motivations

Respondents were asked how much it bothered them to throw out food because it was not eaten, according to a 3-point Likert-type scale including “does not bother me at all,” “bothers me a little,” and “bothers me a lot.” Fifty-two percent reported that discarding food bothered them “a lot”, while 9% reported, “not at all.” In comparison, more respondents were bothered “a lot” by letting the faucet drip (72%) or leaving the lights on (57%). Fewer were bothered “a lot” by discarding rarely-used clothes (48%) or books (33%).

**Fig 1:**
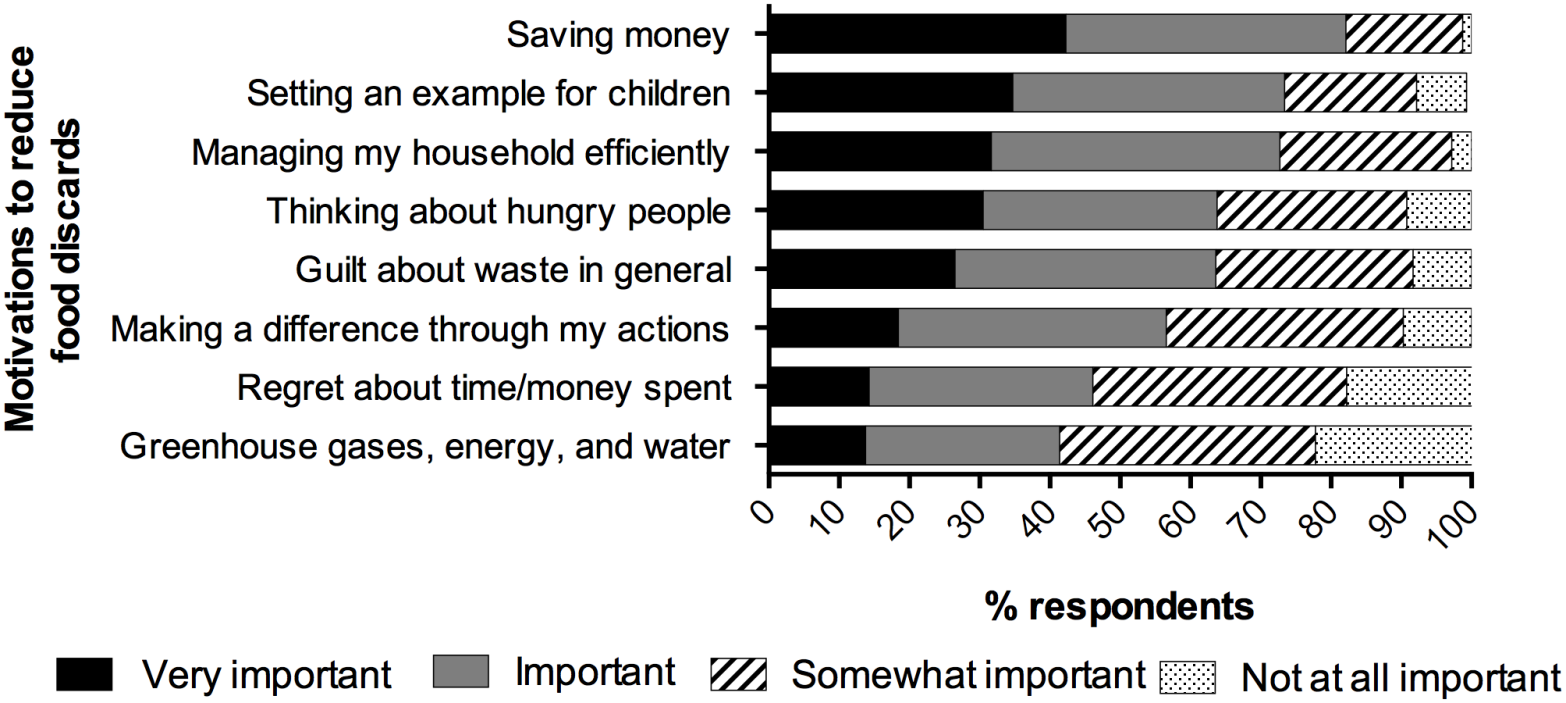
Reported Importance of Motivations to Reduce Food Discards. Responses to four-point Likert-type questions about eight possible motivations for reducing the amount of food discarded. Percentages indicate the proportion of respondents who chose each response, adjusted to 100%.

Respondents were also asked to rate a set of potential motivations for reducing food discards according to a 4-point Likert-type scale of importance, ranging from “not at all important” to “very important.” As shown in Fig 1, the most important motivation was saving money, with setting an example for children coming in second among parents. Notably, 22 percent of respondents said that the environmental concerns of greenhouse gas emissions, energy and water were “not at all important” motivations.

**Fig 2:**
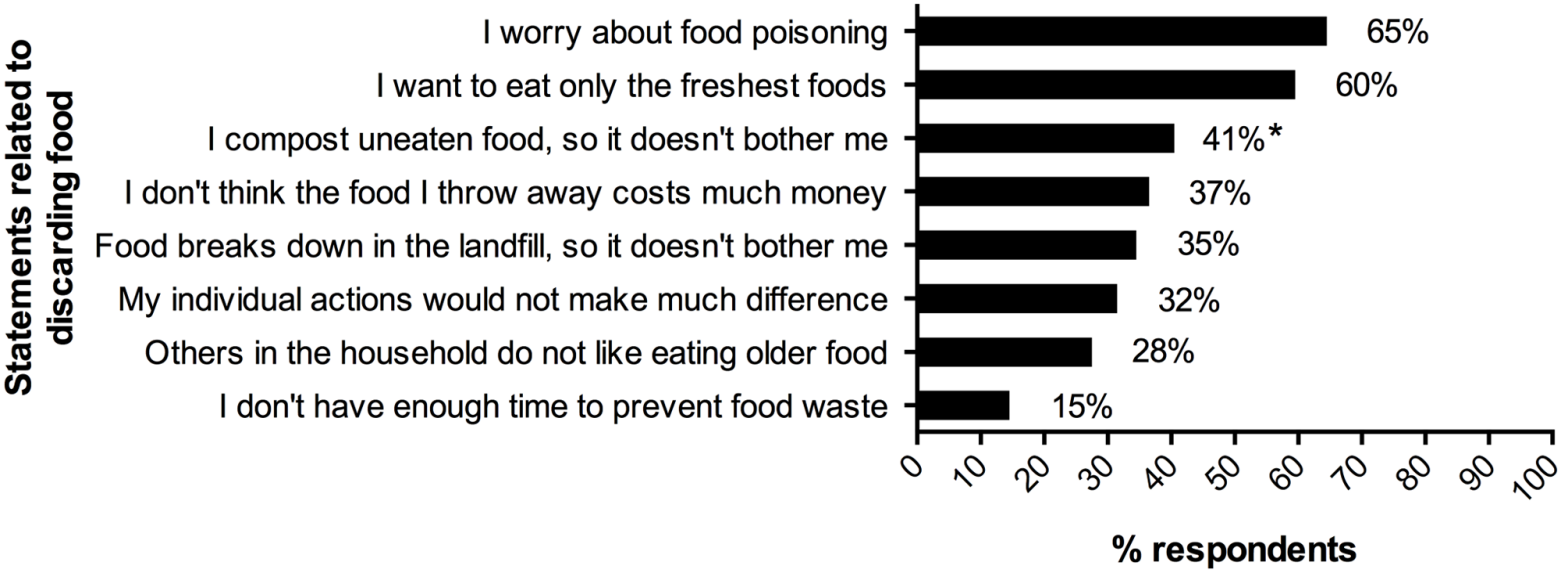
Agreement with Statements Related to Discarding Food. Responses regarding eight possible reasons for discarding food. Percentages indicate the proportion of respondents who chose each response. * Restricted to respondents reporting in a separate question that they compost at least some of their food; percentages for all other motivations reflect the entire sample.

To understand respondents’ reasons for discarding food, they were asked to indicate their agreement or disagreement with a set of statements. Responses suggest the most common reasons were concern about foodborne illness and desire to eat only the freshest food (Fig 2). Among people who reported composting, 41% indicated that because they compost, discarding food does not bother them.

### Behaviors

**Fig 3:**
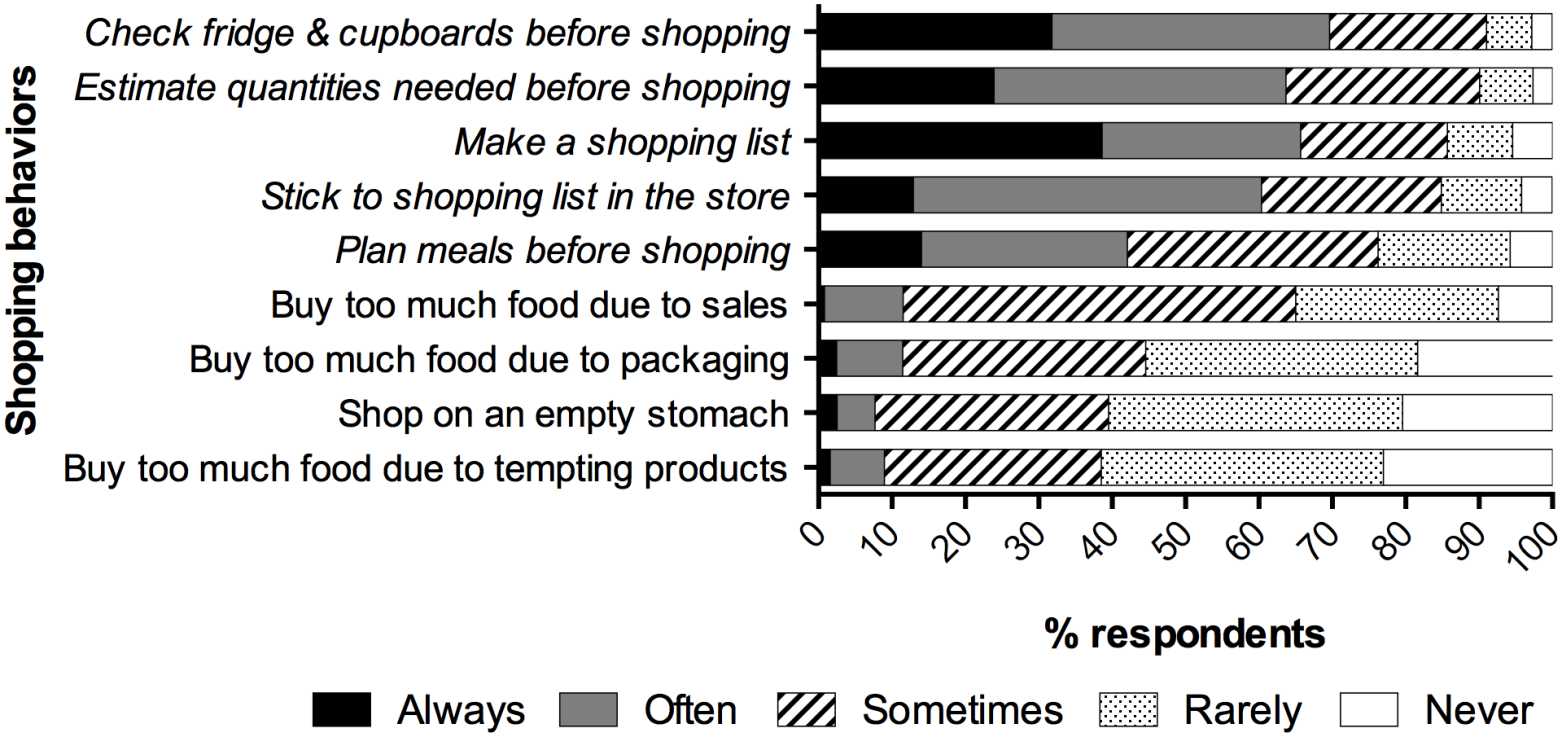
Reported Frequency of Shopping Behaviors. Responses to five-point Likert-type questions about the frequency of performing nine behaviors related to food shopping. Percentages indicate the proportion of respondents who chose each response. Behaviors classified as “food waste reducing” are italicized; behaviors classified as “food waste promoting” are non-italicized.

**Fig 4:**
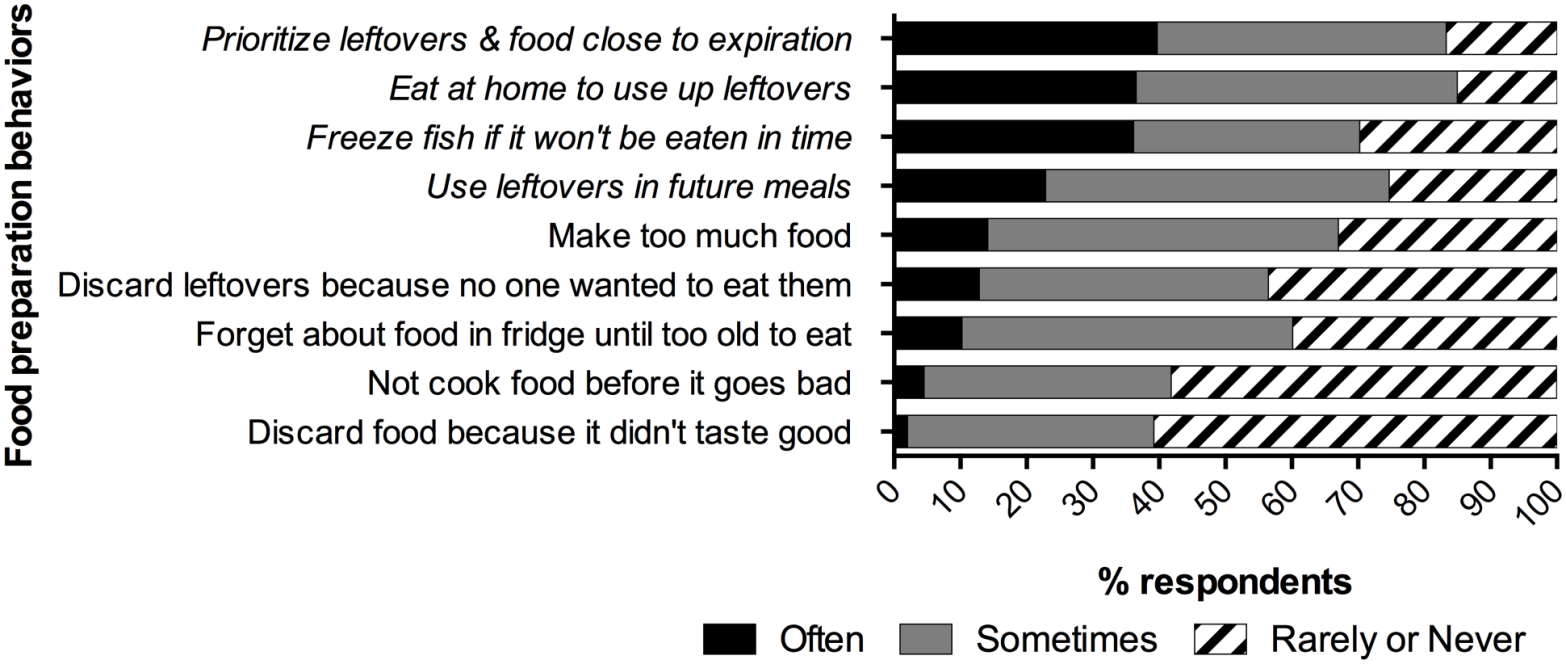
Reported Frequency of Food Preparation Behaviors. Responses to three-point Likert-type questions about the frequency of performing nine behaviors related to food preparation. Percentages indicate the proportion of respondents who chose each response. Behaviors classified as “food waste reducing” are italicized; behaviors classified as “food waste promoting” are non-italicized.

Respondents were asked to indicate the frequency with which they performed a variety of food shopping and food preparation behaviors, presented in random order. Fig 3 presents reported performance of shopping behaviors, and Fig 4 presents food preparation behaviors. We characterize these behaviors as either waste-reducing (italicized) or waste-promoting. The figures depict substantial portions of respondents performing waste-reducing behaviors always or often, and waste-promoting behaviors rarely or never—though there remains considerable space for improvement.

Several questions were asked to gain insight into consumer decision-making about discarding food. First, respondents selected which of five indicators they used when deciding when to throw out milk. Most common was “use my senses,” reported by 72% of respondents; 39% reported using the “use by” date; 22% the “sell by” date [an indicator used by stores, leaving a buffer of time when products still remain high quality]; 18% reported thinking about how long the milk had been opened; and 12% reported thinking about how the milk had been stored (e.g., how long it had been left out). The mean number of indicators used was 1.5 (median 1). In a separate question assessing use of date labels overall, 91% of respondents said they pay attention to date labels. Another aspect of consumer decision-making is willingness to use food that is safe but older. Consumers were asked to move a slider to indicate the maximum percentage brown at which they would accept bananas. The mean response was 40% brown (IQR: 25%, 64%). As shown in Table 2, this decision was significantly associated with gender and household income quintile.

The next set of questions addressed respondents’ current level of effort to reduce the amount of food discarded, and their interest in and perceived difficulty of doing so. Over 1/3 said they exerted “a lot” of effort, with the figure rising to nearly 3/4 when the second of four categories, “a medium amount,” was added. Reported effort in reducing food discards was significantly associated with age and parental status (see Table 2). As for next steps, 23% of respondents reported being very interested in taking action (or additional action) to reduce the amount of food discarded, 65% reported being fairly or somewhat interested, and only 12% reported being not at all interested. When asked how difficult it would be for their household to significantly reduce the amount of food discarded, 43% said easy or very easy, while 16% said difficult or very difficult. Perceived level of difficulty was significantly associated with parental status; 45% of non-parents reported that it would be “very easy” to reduce their household’s discards, compared to only 35% of parents.

### Recommendations for Retail and Restaurants

Respondents were provided with a list of possible changes retailers could make to help reduce household discarding of food (S2 and S3 Figs). The most popular changes respondents said they “would like to see” were more resealable packages (57%), more variety in product sizes (50%), “buy one, get one later” sales (48%), and discounting foods that are over-ripe or near expiration (48%). Respondents were also asked which products they wished were sold in smaller packages. Top responses included baked goods, bagged salad, bread, and meat (43, 41, 39 and 29%, respectively). Frequent write-in responses included milk, fruit, vegetables and canned goods.

Although restaurants could make many changes to reduce discarding of food, some of these changes may be perceived as unacceptable to patrons. Accordingly, respondents were queried about which of a list of changes they would consider acceptable. The leading items were donating excess food (73%), serving smaller portions (61%), taking time to make items to order rather than serving only ready-made items (37%), and providing smaller salad bar plates (30%). Respondents were less accepting of reduced menu variety (15%) and eliminating salad bar trays in favor of plates (8%) (S4 and S5 Figs).

## Discussion

This survey paints a portrait of Americans’ awareness, perceived knowledge, attitudes and behaviors regarding wasted food as of mid-2014, as prevention efforts are beginning to build. Overall, the survey suggests substantial receptivity to waste prevention activities, with a high self-reported baseline level of awareness, knowledge, and positive attitudes; and moderate rates of desired behaviors. There were important commonalities between our findings and those from international surveys, suggesting that there is some benefit to utilizing insights from international research on other aspects of food waste prevention while awaiting further evidence from the U.S. context. That said, there are substantial cross-national differences in culture and society, food system, infrastructure, policy, and geography; shopping patterns differ in terms of the amount of food purchased in a single trip, the number of days between shopping trips, and the amount of food stored in the household. Accordingly, even similar survey responses may mask meaningful differences in determinants of waste that would lead to differing prevention strategies.

We found that Americans perceive themselves as wasting little, with nearly three-quarters reporting that they discard less food than the average American. International surveys also found high percentages of the public self-reporting very low amounts of wasted food, although the percentages in the U.S. seem to be even higher. For example, compared to our finding of 69% reporting discarding 0–10% of food, in the UK in 2007 as food waste activities were developing, 43% of consumers said they discarded “hardly any” or no food [18], and in New South Wales, Australia, 63% said they threw out “very little” food [27]. We also found—as did WRAP in the U.K.—that a majority of respondents reported investing a great deal or a fair amount of effort in minimizing the food discarded (73% in our study; 67% in WRAP’s) [18]. WRAP found that younger age groups reported less effort, while we noted no differences by age group.

Based on what is known about wasted food in the U.S., it is clear that respondents as a group are substantially underreporting their waste levels, and they may also be overreporting their effort levels. It is well-known that surveys are an ineffective tool for assessing waste levels[3], and thus our questions regarding the amount of food wasted were asked only with the intent of understanding respondent perceptions. In-depth research using trash sorting and diaries is needed to gain insights into actual waste, while qualitative and other methods will be more effective for understanding the underlying social phenomena. It is possible that in comparing their own waste to national figures, respondents commonly used judgment heuristics involving anchoring their estimates to known figures. For example, the estimate that 40% of all food is wasted would lead most people to say that they waste less than that [2,3,28]. Regarding effort, we asked only a general question about the amount of effort invested, aimed at drawing a broad-brush picture of respondent perceptions; it would be valuable for follow-up research to ask detailed questions about effort. This survey does not enable assessing the extent to which the above findings reflect a lack of awareness, aspirational reporting, cognitive dissonance, social desirability bias, or biases due to judgment heuristics.

Regardless of the accuracy of these estimates, given the volume of food wasted in the U.S. and lack of attention to the issue thus far, it would be easy for interventionists to assume they should “start from zero.” These self-reports send a message that communications may resonate more if they are framed to recognize people’s view of themselves as engaged and knowledgeable.

Among reported motivations for reducing food discards, saving money topped the list, with setting an example for children second (a motivation that did not appear in other surveys reviewed), followed by other concerns related either to efficiency or guilt. These findings were similar to those found in other studies [3,18,29,30]. Environmental concerns ranked last, with just over 10% of respondents rating them as “very important.” Surveys in other countries have also found environmental concerns to rank behind others—though perhaps not with quite as low priority as we found. For example, 20% of respondents indicated a priority on environmental concerns in both U.K. and the U.S. Sustainable America survey; and in Canada, when asked to characterize the problem of food waste, “an environmental problem” (68%) came in third behind “social” (83%) and “economic” (72%) problem. [3,18,19,29,30]. Many interventionists and food waste prevention organizations are driven by environmental concerns, however, our finding suggests that their work might have more resonance if it focused on budget and other factors that more strongly motivate American consumers. That said, we note that it is possible that if consumers were aware of the actual cost of the consumer portion of food that is wasted (an average of $371 per capita per year according to the USDA’s estimate), it might not be sufficient to motivate most non-low income consumers.

It is an open question why respondents placed so little priority on environmental concerns; possibilities include a lack of knowledge, a desire to avoid thinking about the environmental consequences of wasting food, and a weighting of “altruistic” environmental factors as less motivational than the more personally relevant concerns of saving money and aligning one’s behaviors and moral attitudes (particularly if the environmental impacts are not viewed as significant.) Other research has found that Americans have low levels of environmental knowledge (with considerable variation across topics) [31], and also that despite reported concerns about the environment, levels of action are low [32]. Knowledge and concern are commonly found to be important but insufficient precursors to pro-environmental behaviors [33,34]. The relationship may be even more complex in the case of behaviors that can alternatively be motivated by non-environmental concerns. These findings suggest support for all of these possibilities, and a likely need both for education about environmental impacts of wasted food, and for more sophisticated strategies aimed at addressing this discordance.

Based on our findings, there are several critical areas for further work. First, concern about foodborne illness was the most common reason given for discarding food, yet, for most foods, contamination and spending too long at the wrong temperature are key to risk, rather than primarily food age—although certainly those factors intertwine [35]. There is thus a great need for clearer food safety guidance, for different foods, presented in the joint context of waste prevention and food safety. There is also a need for improved understanding of how Americans make decisions about when to discard foods, including their level of knowledge and their rational thought processes, as well as their implicit, unconscious and habitual attitudes and behaviors. Additionally, there is a need for a nationally harmonized and well-communicated expiration date label system to reduce consumer confusion and resultant unnecessary wastage [18,19,36–39].

Second, a majority of respondents reported that they only wanted to eat the freshest food. While humans may have a natural preference for freshness, this concept has also been heavily promoted by health advocates, cooking shows, local food supporters and others as a strategy for making produce more palatable, to add economic value to fresh and local foods, and to enhance both enjoyment and status [40]. Food waste prevention must incorporate efforts to expand the acceptability of still-good produce and other foods that are older and/or less aesthetically pleasing, and those nearing their expiration dates. Such items can be sold at a discount. There is a substantial space where food may appear less attractive but remains healthy, and when well-prepared, equally palatable.

To improve our ability to intervene and target appropriate interventions, there is a need for research drilling deeper into every one of these reasons for waste. For example, in the case of food safety, *which* foods are consumers discarding for food safety reasons, and how much of that legitimately *should* be discarded? What are the key reasons for food remaining uneaten long enough to become unsafe? What drives incorrect perceptions of food safety?

Communications research can provide additional depth of understanding regarding what messages motivate actual behavior change, and what messages influence people to prioritize food safety, even as they increase waste prevention. There is a related need to probe more deeply into consumers’ self-reported relatively high level of knowledge about wasted food; understanding the extent to which they are actually knowledgeable can help inform whether interventions ought to assume people are knowledgeable, versus merely catering to their self-perceived high level of knowledge by avoiding messages that treat them as new to the issue.

Other areas indicated for additional communication include: the cost of wasted food, because that speaks to people’s concerns; the environmental impact, because of the possible low awareness; and promoting food-related behaviors that may take place far in advance of the waste and that may not be conceptually associated with waste in people’s minds[3]. Further, the concept that composting food still represents waste (albeit a preferable alternative to sending uneaten food to landfills) should be highlighted, as a significant portion of composters (41%) reported that discarding food does not bother them *because* they compost (studies elsewhere have also highlighted similar concerns, although commonly at lower levels than we found) [18,29,38,41].

In addressing consumer-level waste of food, it is easy to assume a personal responsibility frame, with most efforts focused on education and communication[4]. Extensive evidence from public health and related fields shows limitations of the individual responsibility frame [42], and highlights that shame and blame can be counterproductive [43]. Consumer education and behavior change interventions must be complemented by approaches making use of entrepreneurial, policy, economic, behavioral economic, and other tools. Indeed, our findings suggest consumer interest in and acceptability of several changes that could be made by the food industry at little cost or even at profit, including smaller packages, donating food, smaller portions, discounts for less aesthetic foods, and making food to order. Behavioral economics approaches would additionally be valuable, to address shopping patterns and their influence on the kinds and amounts of food purchased. There is a need for additional research to understand the tradeoffs for consumers in increased unit price of smaller packages versus cost of food that would otherwise be wasted. While more environmentally-friendly packaging is always desirable, some evidence suggests that for many foods and packaging types, the environmental impacts of extra packaging needed to create smaller sizes may be less than that of the food that would be wasted, although again, additional research would be valuable [44,45].

Overall, this survey found few differences in reported waste or other factors based on race, education, or rural/urban status. Modest differences by age and parental status suggest that older people report less waste of food, and parents of children under 18 report feeling less knowledgeable about how to reduce food discards in their households. In the latter case, it is interesting to note one recent study finding that while adults consumed 89–92% of what they served themselves, children consumed only an average of 59%, suggesting that eating less of one’s food may be a normal child behavior—albeit one frustrating to many parents [46]. We identified some differences based on income that reached statistical significance, however, there were not clear linear relationships leading to readily interpretable messages. International surveys have varied considerably regarding which demographic predictors rose to the top. For example, a survey in the U.K. identified age, status as a parent, income and gender [18] as predictors of wasted food, while one in Finland found household size and type, gender of grocery shopper, valuation of low prices, and views of potential to reduce food waste among the top predictors [41]. Unlike some other studies, ours did not observe a linear relationship between income and reported level of food wasted; additional inquiry is needed in the U.S. and internationally to gain insight into that relationship.

### Strengths and Limitations

As the first national consumer survey focused on wasted food (one prior survey included a set of questions on the topic), this study provides important insights that can guide intervention and serve as a baseline for activities to address such waste in the U.S. Effort was made to improve national representation through the use of stratification and clustering to develop the survey firm’s database of respondents and application of post-survey weights, though we do note that the unweighted survey sample underrepresented those in the lowest income quintile and minorities. An additional strength is that questions used in other surveys were incorporated for comparability. The chief limitation is that surveys cannot provide information about actual behaviors or attitudes, only reported ones. The survey used anonymous administration and randomized question order to reduce bias [47]. Survey administration was slightly expedited, due to anticipated food waste communication activities on Earth Day; those who respond to a survey soon after being contacted may differ in unmeasured ways from those responding later.

## Conclusions

Consumer waste of food in the U.S. represents a powerful quintuple threat; reducing it may improve food security, nutrition, budgets, environment and public health. Evidence from the U.K. suggests that multifaceted interventions combined with research can lead to substantial reductions in a short time. This survey, the first known nationally-representative consumer survey focused on wasted food in the U.S., provides essential data to assist those developing interventions using education, changes in business practices, and policy, as well as baseline data against which to measure effectiveness. It suggests approaches including avoiding treating consumers as if they are completely new to the issue of wasted food, framing messages focused on budgets, and providing smaller portions. In addition, there is a need to sharpen our messages about food safety and freshness in order to expand acceptability of those foods that may be less attractive or nearing their expiration dates but are unlikely to be hazardous, while avoiding waste prevention messaging that could increase food safety hazards. The survey also suggests opportunities to shift business practices to support consumer-desired changes that prevent waste. It highlights critical research gaps, particularly in better understanding the actual behaviors underlying the survey responses provided. Finally, it provides reason for optimism about waste prevention in the U.S.—because respondents are concerned about wasted food, and are interested in taking further action.

## Supporting Information

S1 Fig Desired information to assist in reducing food discards. (TIFF)

S2 Fig Changes retailers could make to reduce food discards. (TIFF)

S3 Fig Products for which respondents want smaller packages. (TIFF)

S4 Fig Potentially unaccepted restaurant changes that respondents consider acceptable. (TIFF)

S5 Fig Restaurant changes that would be helpful. (TIFF)

S1 File Survey questionnaire. (DOCX)

S2 File Survey data. (XLSX)
