# Supplementary figures and images for: Wasted Food: U.S. Consumers' Reported Awareness, Attitudes, and Behaviors
Source: PLoS One. 2015 Jun 10;10(6):e0127881. doi: 10.1371/journal.pone.0127881 (PMC4465675; doi:10.1371/journal.pone.0127881)

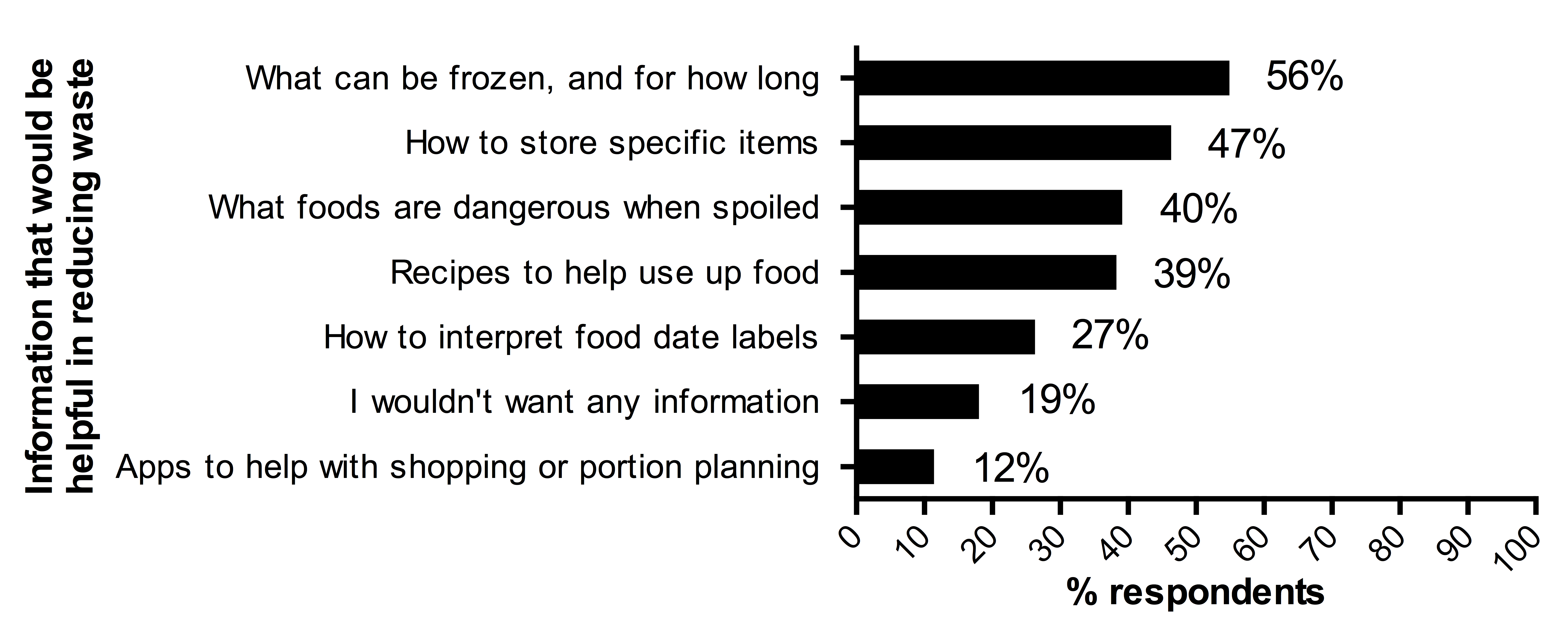

Supplement: S1 Fig — (TIFF) [file pone.0127881.s001.tiff]

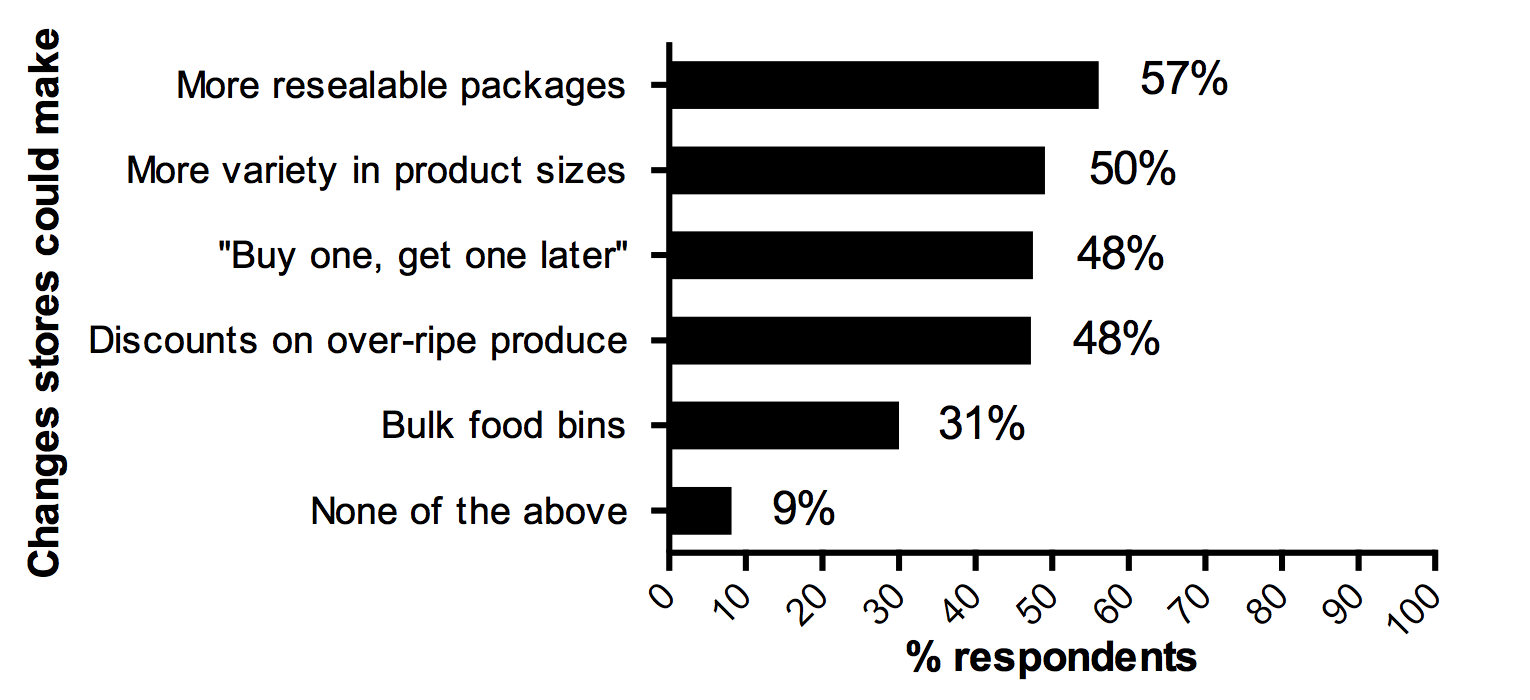

Supplement: S2 Fig — (TIFF) [file pone.0127881.s002.tiff]

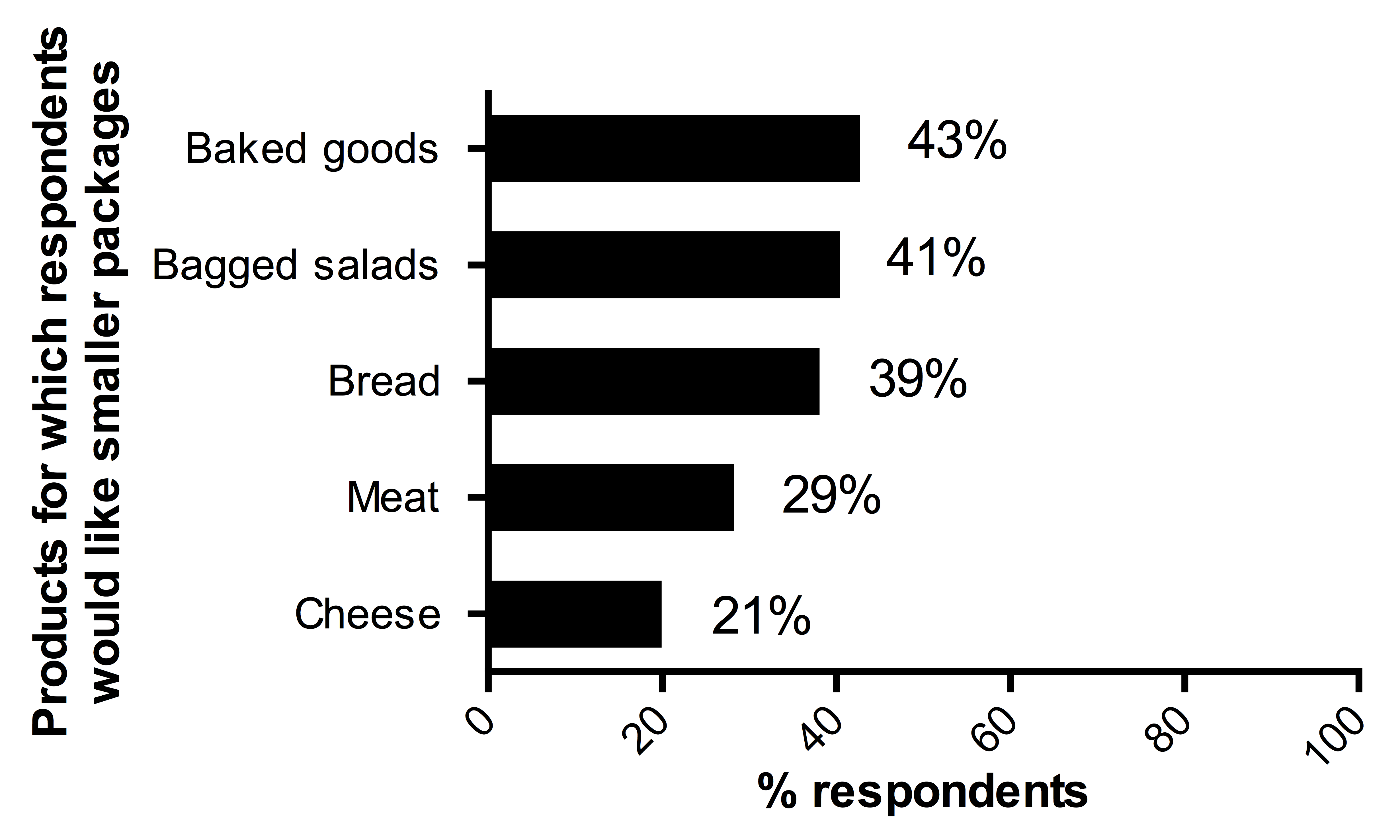

Supplement: S3 Fig — (TIFF) [file pone.0127881.s003.tiff]

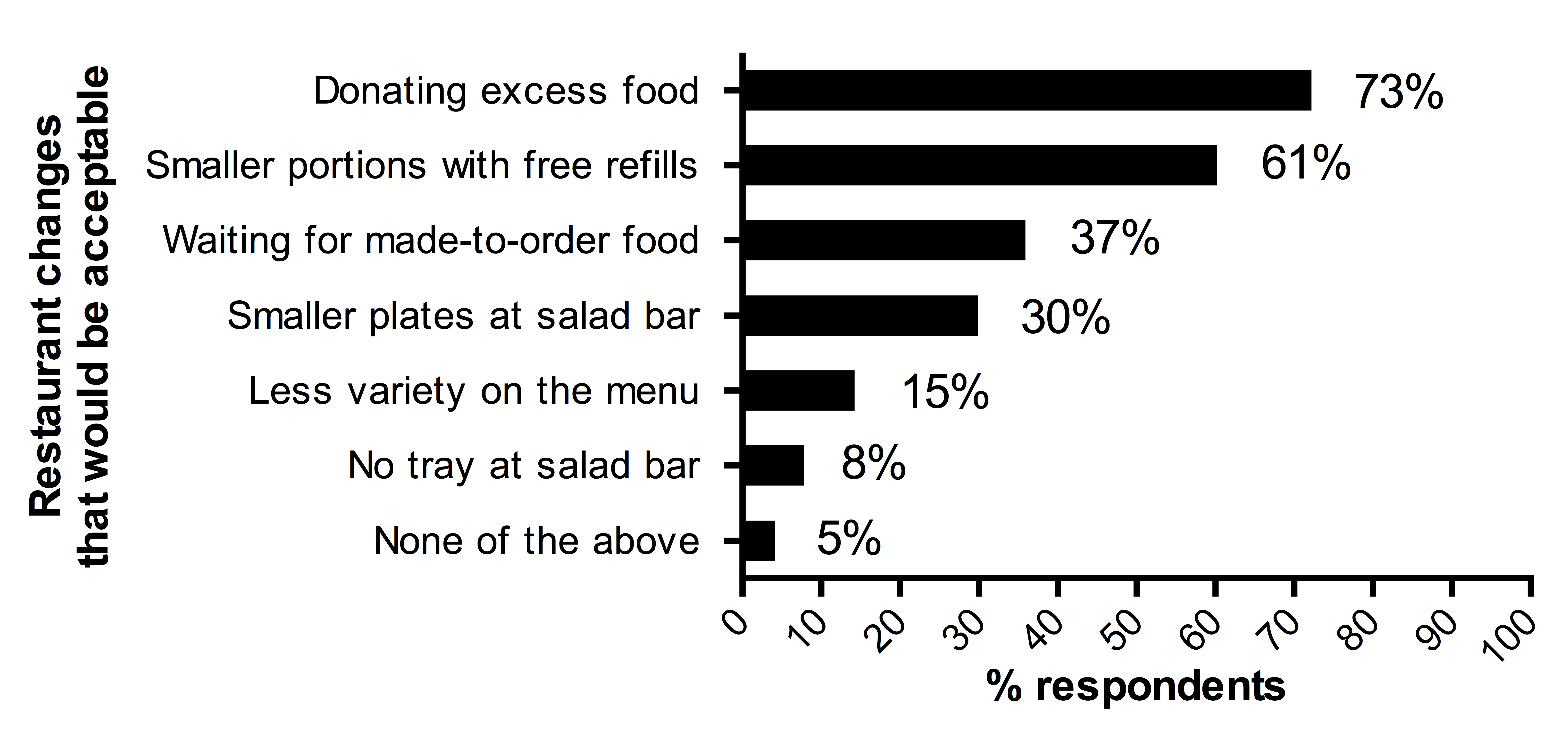

Supplement: S4 Fig — (TIFF) [file pone.0127881.s004.tiff]

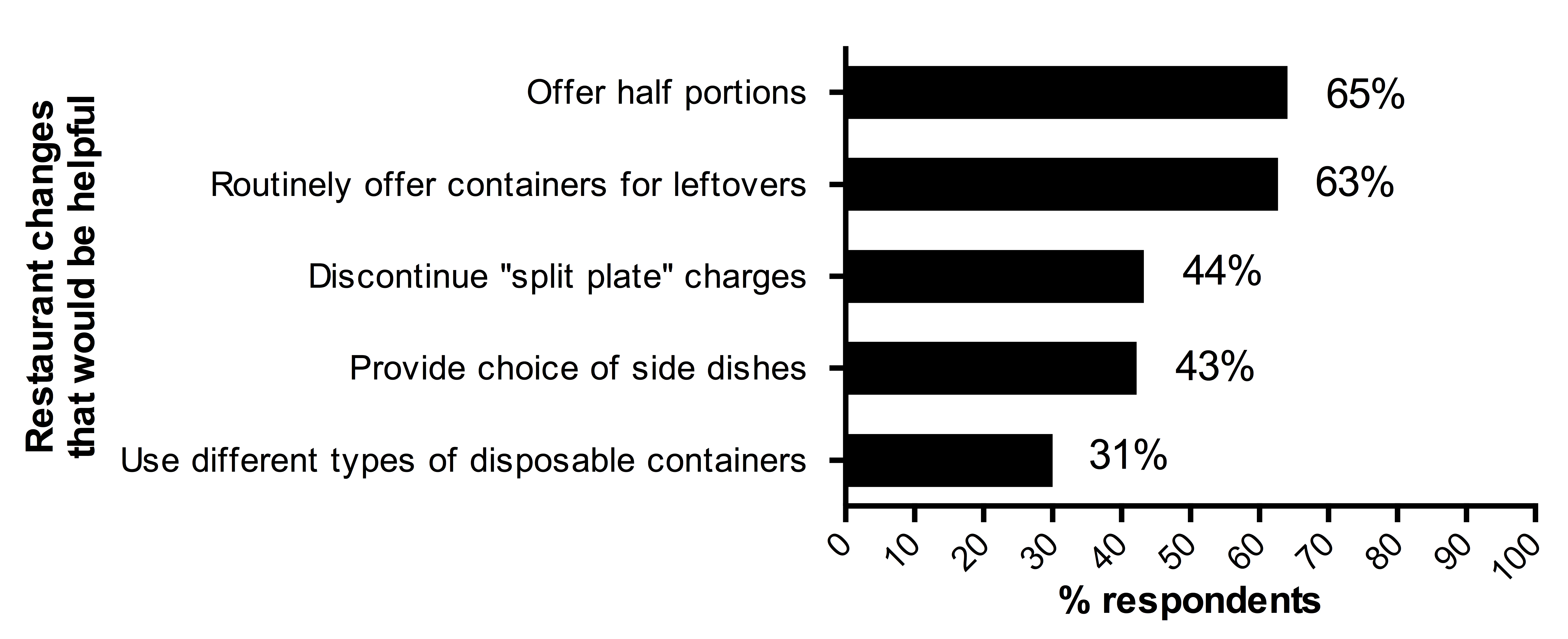

Supplement: S5 Fig — (TIFF) [file pone.0127881.s005.tiff]
