# Supplementary material for: Wasted Food: U.S. Consumers' Reported Awareness, Attitudes, and Behaviors
Source: PLoS One. 2015 Jun 10;10(6):e0127881. doi: 10.1371/journal.pone.0127881 (PMC4465675; doi:10.1371/journal.pone.0127881)
Supplement: S1 File — (DOCX) [file pone.0127881.s006.docx]

Appendix A: Questionnaire – Main Survey

Food Waste

[DISPLAY ON SAME SCREEN AS Q1]

Thank you for agreeing to complete this survey! We are interested in your opinions about a number of household issues.

[SP]

Q1. When it comes to cooking in your household, would you say…

1. You do all or most of it

3. You do about half of it

4. Someone else does all or most of it

[SP] [SHOW HALF OF RESPONDENTS ANSWER OPTIONS AS IS AND OTHER HALF IN REVERSE ORDER]

Q2. How much do you like cooking?

1 Hate cooking

2 Dislike cooking

3 Neither like nor dislike cooking

4 Like cooking

5 Love cooking

[SP]

Q3. How frequently do you (or a household member) cook dinner?

1 Daily

2 Several times a week

3 Once a week

4 Less than once a week

[SP]

Q4. When it comes to organizing the refrigerator and cupboards, would you say that you…?

1. Do all or most of it

2. Do about half of it

3. Someone else does all or most of it

[SP]

Q4a. When it comes to shopping in your household, would you say…

1. You do all or most of it

3. You do about half of it

4. Someone else does all or most of it

[NUMBER BOX; RANGE 0 – 1000000 (PUT A DOLLAR SIGN IN FRONT OF NUMBER BOX]

Q4b. About how much money does your household spend on food and beverages each week, including foods eaten at home and away from home?

(Give your best estimate)

$________

[GRID; SP ACROSS; RANDOMIZE]

Q9. The next question is about household goods and services that are not used as intended.

How much does each of the following bother you?

Does not bother me at all; Bothers me a little; Bothers me a lot

A. Leaving the lights on in an empty room

B. Letting the faucet drip

C. Throwing out food from the fridge because it wasn’t eaten

D. Buying books that are rarely or never used

E. Buying clothes that are rarely or never worn

The rest of this survey will focus on the issue of food and beverages intended for human

consumption that go unused and are discarded in one way or another (thrown out, put in a

garbage disposal, fed to animals, composted, never harvested or sold, or otherwise not eaten by humans). This includes food or beverages that have spoiled.

[SP]

Q10. In the past year, have you seen or heard anything in the news, social media, or elsewhere about the issue of food that is thrown out or otherwise not eaten by humans? (Sometimes referred to as “wasted food”)

1. Yes

2. No

3. Not sure

[IF Q10=1]

[MP; RANDOMIZE OPTIONS 1-4]

[SHOW ON SAME SCREEN AS Q10 BUT KEEP HIDDEN UNTIL Q10 IS ANSWERED]

Q11. What did you see or hear about these issues?

1. Information about how much food is thrown out

2. Information about how to avoid throwing out food

3. Information about food expiration date labels

4. Information about composting or ways to dispose of food that is thrown out

5. Something else (please specify) [TEXT]

[SP]

Q12. In the past year, have you sought out any information about ways to reduce the amount of food you throw away?

1. Yes

2. No

[SP]

[SHOW HALF OF RESPONDENTS ANSWER OPTIONS AS IS AND OTHER HALF IN REVERSE ORDER]

Q13. Of all the food that is produced for human consumption in the U.S. each year, about what percent would you guess is discarded or not eaten by humans? (Do not include inedible food scraps and bones in your estimate).

1. 5%

2. 10%

3. 20%

4. 40%

5. 60%

[DISPLAY ON SAME SCREEN AS Q15]

The following questions are about the ways that the people in your household buy and use food. Please think about your whole household when answering.

[GRID; SP ACROSS; RANDOMIZE]

Q15. Think about the last week in your household. Describe the amount of each type of food below that you and other members of your household threw away.

None, Hardly any, Some, A fair amount, A lot, We don’t eat this food

A. Fruit/vegetables

B. Bread

C. Meat

D. Milk

E. Packaged foods meant to be stored outside the fridge (like breakfast cereal, cookies,

canned vegetables)

F. Home-made meals

[DISPLAY ON SAME SCREEN AS Q16A]

The following two questions refer to ALL types of food together (including fruits/vegetables, breads, milk, cheese, meat, fish, processed foods, and meals prepared at home and purchased).

[SP]

[SHOW HALF OF RESPONDENTS ANSWER OPTIONS AS IS AND OTHER HALF IN REVERSE ORDER]

Q16a. About what percent of all the food that comes into your household is later thrown away? Give your best estimate.

1. 0%

2. 10%

3. 20%

4. 30%

5. 40%

6. 50%

7. More than 50%

[SP]

[SHOW ON SAME SCREEN AS Q16A]

Q16b. How much of this (throwing out food) could have been avoided?

1. None

2. A little

3. A fair amount

4. A lot

[SP]

[SHOW HALF OF RESPONDENTS ANSWER OPTIONS AS IS AND OTHER HALF IN REVERSE ORDER]

Q18. Do you think the amount of food you throw out is (More, The same, Less) than

the average American?

1. More

2. The same

3. Less

[DISPLAY ON SAME SCREEN AS Q20]

The next few questions focus on food shopping for your household. Even if you are not your household’s main shopper, please answer these as you think the main shopper would.

[GRID; SP ACROSS; RANDOMIZE]

Q20. How often do you do the following shopping-related activities?

Never Rarely Sometimes Often Always Not applicable

A. Make a shopping list

B. Check to see what is in your refrigerator and cupboards before you go shopping

C. Plan your meals before shopping

D. Estimate how much of various items you will need before shopping

E. Stick to your shopping list in the store

F. Get tempted in the store and buy appealing products that may not be eaten

G. Due to the way food is packaged, buy food in larger packages than you are likely to use before it spoils

H. Due to sales, buy more food than you are likely to use before it spoils

I. Shop on an empty stomach

[GRID; SP ACROSS; RANDOMIZE]

Q45. Following are some statements about fresh, processed, canned and frozen foods. Please

indicate the extent to which you agree or disagree when it comes to your household.

Agree strongly; Agree; Neither agree nor disagree; Disagree; Disagree strongly; Not applicable

A. Because fresh foods go bad more quickly than other foods, we buy more processed food than we would otherwise.

B. Because fresh foods go bad more quickly than other foods, we buy more canned or

frozen food than we would otherwise.

C. Fresh foods go bad in my household because people prefer other foods.

D. [IF Q45C=1-3 – SHOW JUST BELOW Q45C] Because people in my household prefer other

foods compared to fresh foods, we buy less fresh food than we would otherwise.

[GRID; SP ACROSS; RANDOMIZE]

Q21. Are there products you wish were sold in smaller packages?

Yes; No; I don’t buy/eat this food

A. Bread

B. Baked goods

C. Meat

D. Bagged salads

E. Cheese

F. Other (please specify) [TEXT]

[SP]

Q22a. Nutritional advice from many sources tells us to eat more fruits and vegetables. How does this advice affect the amount of fruits and vegetables your household buys?

We tend to buy more fruits and vegetables than we would otherwise.

1. Agree

2. Disagree

[IF Q22A=1]

[SHOW ON SAME SCREEN AS Q22B BUT HIDE UNTIL Q22A IS ANSWERED]

Q22b. Sometimes these “extra” fruits and vegetables go bad before we can eat them.

1. Agree

2. Disagree

[SP]

Q23a. Are you aware of the US government’s “My Plate” dietary guidelines?

1. Yes

2. No

3. Not sure

21

[IF Q23A=1]

[SP]

Q23b. Are you aware that the “My Plate” dietary guidelines advise that we fill half our plates

with fruits and vegetables at each meal?

1. Yes

2. No

3. Not sure

[IF Q23B=1]

[SP]

Q23c. How do the “My Plate” dietary guidelines affect the amount of fruits and vegetables your household buys – above and beyond the choices you might make based on your knowledge of nutrition?

Because of the “My Plate” dietary guidelines, we tend to buy more fruits and vegetables than we would otherwise

1. Agree

2. Disagree

[IF Q23C=1]

[SHOW ON SAME SCREEN AS Q23C BUT HIDE UNTIL Q23C IS ANSWERED]

Q23d. Sometimes these “extra” fruits and vegetables go bad before we can eat them.

1. Agree

2. Disagree

[MP]

[RANDOMIZE OPTIONS 1-7]

Q32. Following are some changes stores can make to reduce the amount of food that gets

thrown out. Which of the following would you like to see?

1 "Bulk Food" bins where I can scoop out exactly the amount I need (for instance, for

spices, flour, sugar, etc.)

2 “Buy one, get one later” offers, rather than “buy one, get one free.”

3 Discounts on over-ripe produce or food near its expiration date

4 More variety in product sizes

5 More foods offered in resealable packages

6 Other (please specify) [TEXT]

7 None of the above [SP]

[DISPLAY ON SAME SCREEN AS Q24 (FOR BOTH THE FIRST SCREEN AND SECOND SCREEN)]

The next set of questions focuses on how your household uses food.

[GRID; SP ACROSS; RANDOMIZE]

[SPLIT ACROSS TWO SCREENS]

Q24. How often do you and/or your household members do the following with food?

Rarely or never; Sometimes; Often; Not applicable

A. Freeze fish if you think you will not be able to eat it in time

B. Use leftovers or food scraps as ingredients in future meals

C. Prioritize eating leftovers and foods close to expiration or spoilage

D. Eat at home if you have food to use up, even if you feel like going out

E. Make too much food

F. Eat when you are not hungry to avoid wasting food

G. Not cook food before it goes bad

H. Throw away leftovers because no one wanted to eat them

I. Throw away food because a new recipe or unfamiliar item did not taste good

J. Forget about items in your fridge until they are too old to eat

[SP]

Q25a. Do you pay attention to date labels on food, such as “use by,” “sell by” and “best before”?

1. Yes

2. No

[SP]

Q25b. Do you make different decisions about whether to eat foods depending on whether the date label says, “use by,” “sell by,” or “best before”?

1. Yes

2. No

[MP]

Q29a. How do you decide when to throw away milk?

1. Look at “sell by” date

2. Look at “use by” date

3. Use my senses (smell, taste, or look at it)

4. Think about how long it has been open or in my home

5. Think about how it has been stored (e.g., whether it got left out of the refrigerator)

6. Other (please specify) [TEXT]

[SLIDER; RANGE: 0% TO 100%]

Q28. In deciding whether to eat a banana, how much “brown” will you accept?

Please click on the slider bar below to indicate your answer. You can slide the indicator to the position that best describes your opinion. If the indicator does not work, you can enter a number in the number box.

[SLIDER BAR HERE – show labels for 0% (No brown at all), 50% (Half brown), and 100% (All brown)

[MP]

[RANDOMIZE OPTIONS 1-5]

Q33. When you or other household members dispose of food in your home, where does it go?

1 Trash

2 Garbage disposal in sink

3 Home compost bin

4 Compost pickup

5 Feed to pet/livestock

6 Other (please specify)[TEXT]

[DISPLAY ON SAME SCREEN WITH Q30]

The next two questions are about food discarded in restaurants.

[MP]

[RANDOMIZE OPTIONS 1-5]

Q30. There are many ways restaurants can help patrons reduce the amount of food they throw

out. Which of the following would you consider most helpful?

1 Provide choices of side dishes

2 Discontinue “split plate” charges (extra fees when two people split a dish)

3 Offer half portions

4 Use different types of disposable containers for leftovers

5 Routinely offer containers for leftovers

6 Other (please specify) [TEXT]

[MP]

[RANDOMIZE OPTIONS 1-5]

Q31. Restaurants can also take important steps “behind the scenes” to reduce the amount of food they throw out, but many of these will have some impact on consumers. Which of the following changes in a restaurant would you consider acceptable?

1 Less variety on the menu

2 Smaller portion size (with free refills)

3 Smaller plates at salad bar

4 No tray at salad bar

5 Extra time for making items to order (versus having them ready-made)

6 Donating excess food

7 None of the above [SP]

[DISPLAY ON SAME SCREEN WITH Q36]

The next few questions are about the reasons food does and does not get thrown out.

[GRID; ACROSS; RANDOMIZE A - J]

Q36. Following are some statements about throwing away food. Please indicate whether you agree or disagree with each of them.

Agree; Disagree; Don’t know; Not applicable

A. I don’t have enough time for the actions that would prevent throwing out food

B. I don’t think the amount of food I throw away costs me much money

C. My household’s uneaten food is composted, so throwing it out does not bother me

D. I want to eat only the freshest foods

E. I sometimes throw out food because I worry about food poisoning

F. Food is natural and breaks down in the landfill, so throwing it out does not bother me

G. Given the amount of food that is thrown out in this country, my individual actions would not make much difference

H. Other people in my household don’t like when I try to use up older food

I. Other (please specify) [TEXT]

[GRID; SP ACROSS; RANDOMIZE]

[REPEAT COLUMN HEADER AFTER ITEM 5 INSTEAD OF 6]

Q35. How important are the following in motivating you to reduce the amount of food your household discards?

Not at all important; Somewhat important; Important; Very important; Not applicable

A. Thinking about people without enough to eat

B. Thinking about the possibility of saving money

C. Feeling regret about time spent shopping, storing, or preparing food not eaten

D. Wanting to manage my home efficiently

E. Feeling guilty about waste in general

F. Thinking about the greenhouse gases, energy and water resources it took to get the

food to my plate

G. Thinking about the idea that I can make a difference through my actions

H. Wanting to set an example for my children

I. Other (please specify) [TEXT]

[SP]

Q19. How much effort do you currently make, to minimize the amount of food you throw

away?

1. None

2. A little

3. A medium amount

4. A lot

[SP]

Q34. How interested are you in taking [IF Q19=2-4 INSERT: additional] action to reduce the amount of food your household wastes or discards?

1. Not at all interested

2. Somewhat interested

3. Fairly interested

4. Very interested

[SP]

Q37a. Imagine your household decided to significantly reduce the amount of food that gets thrown out. How difficult would it be to do that?

1. Very difficult

2. Difficult

3. Neither difficult nor easy

4. Easy

5. Very easy

[SP]

Q37c. How knowledgeable do you feel you are about how to reduce the amount of food you throw out?

1. Very knowledgeable

2. Fairly knowledgeable

3. Somewhat knowledgeable

4. Not very knowledgeable

[MP; RANDOMIZE OPTIONS 1-6]

Q38. What information, if any, would be helpful in reducing the amount of food your household throws out?

Information about…

1. How to store specific items

2. How to interpret food date labels (use by, sell by, etc.)

3. What can be frozen and for how long

4. What foods are dangerous when spoiled versus simply distasteful

5. Recipes to help you use up food

6. Apps to help with shopping or portion planning

7. Other information (please specify) [TEXT]

8. I wouldn't want any information [SP]

[DISPLAY ON SAME SCREEN AS Q5]

Following are some additional background questions about you and, in some cases, your

household.

[SP]

Q5. Do you or members of your household shop in a farmers market during the local growing season?

1. Rarely or never

2. Sometimes

3. Often

[SP]

Q8. Have you or members of your household ever lived on a farm or been otherwise involved in growing food, including gardening?

1 Yes

2 No

3 Don’t know

[SP]

[IF Q8=1]

Q7. Do you or members of your household currently grow fruits, vegetables or other food in a home or community garden?

1 Yes

2 No

[SP]

Q39. Do you participate in a community supported agriculture (CSA) program (e.g., a program where you pay to receive weekly distributions of food from a farm)?

1 Yes

2 No

[IF Q39=1]

[SP]

Q40. To what extent do you find yourself throwing away uneaten items in your CSA share (e.g., excess greens, or unknown vegetables)?

1 Not at all

2 A little

3 Some

4 A lot

[SP]

Q41. Does either of the following labels describe your eating patterns: vegetarian, vegan?

1. Yes

2. No

[SP]

Q42. Were you OR either of your parents born outside the US?

1 Yes

2 No

[IF Q42=1]

[SMALL TEXT BOX]

Q43. Please indicate your or your parent(s)’ country/countries of origin.

[IF XWASTE=1 AND PPMARIT = 1 OR 6]

[SP]

Q46. Which statement best describes your spouse or partner’s current employment status?

1 Working for pay – full time

2 Working for pay – part time

[SPACE]

3 Not working for pay – stay at home parent, between jobs, retired, etc.

4 Not working for pay – due to disability

[SPACE]

5 Other (please specify) [TEXT] ...............................

[SP]

Q44. Describe your current ability to meet your basic needs:

1. I have plenty and lots to share

2. I have plenty and some to share

3. I have enough

4. I have just enough if I stretch my resources

5. I need some help

[STANDARD CLOSE]

Supplemental Variables

Variable Name Variable Description

CASEID Case Identification Number

WEIGHT Post-stratification weights - Total qualified respondents

TM_START Interview start time

TM_FINISH Interview finish time

DURATION Duration of interview in minutes

XWASTE Data Only Variable : Parent of child 0-17

PPAGE Age

PPAGECAT Age - 7 Categories

PPAGECT4 Age - 4 Categories

PPEDUC Education (Highest Degree Received)

PPEDUCAT Education (Categorical)

PPETHM Race / Ethnicity

PPGENDER Gender

PPHHHEAD Household Head

PPHHSIZE Household Size

PPHOUSE Housing Type

PPINCIMP Household Income

PPMARIT Marital Status

PPMSACAT MSA Status

PPREG4 Region 4 - Based on State of Residence

PPREG9 Region 9 - Based on State of Residence

PPRENT Ownership Status of Living Quarters

PPSTATEN State

PPT01 Presence of Household Members - Children 0 - 2

PPT25 Presence of Household Members - Children 2 - 5

PPT612 Presence of Household Members - Children 6 - 12

PPT1317 Presence of Household Members - Children 13 - 17

PPT18OV Presence of Household Members - Adults 18+

6

PPWORK Current Employment Status

PPNET Household Internet Access
